# Association between friend gatherings and leisure-time exercise among Chinese adults

**DOI:** 10.3389/fpubh.2026.1909863

**Published:** 2026-08-07

**Authors:** Wenfeng Zhu, Yi Lin, Banghui Hong

**Affiliations:** 1 School of Physical Education, Guizhou Normal University, Guiyang, China; 2 College of Physical Education and Sport Science, Fujian Normal University, Fuzhou, China

**Keywords:** active leisure, China, Chinese General Social Survey, friend gatherings, friendship-oriented leisure socializing, leisure-time exercise, physical activity, social capital

## Abstract

**Introduction:**

Broad social-capital measures may obscure the concrete relationships through which leisure-time exercise is organized. This study examined whether leisure-time exercise among Chinese adults is more strongly associated with friend gatherings than with neighborhood interaction, generalized trust, or perceived fairness.

**Methods:**

We pooled nine anonymous repeated cross-sectional waves of the Chinese General Social Survey from 2010 to 2023. Friend gatherings were treated as a proxy for friendship-oriented leisure socializing, not as direct evidence that respondents exercised with friends. Standardized OLS models included socioeconomic and demographic covariates, survey-year fixed effects, and harmonized province fixed effects.

**Results:**

In the primary social-contrast model, friend gatherings showed the largest social-relational association with exercise (standardized beta = 0.149), exceeding the coefficients for neighborhood interaction, generalized trust, and perceived fairness (all coefficient-comparison *p* < 0.001). The association remained positive after adjustment for other leisure activities (beta = 0.081, *p* < 0.001), and robustness analyses supported the same bounded interpretation. Boundary analyses did not show that the pattern was reducible to venue/gym exercise, and supplementary health-profile analyses did not identify exercise-friendship health synergy.

**Discussion:**

Leisure-time exercise in China co-patterns with friendship-oriented leisure socializing more strongly than with broader social-capital attitudes or neighborhood contact. These findings are associative and do not establish causation or direct exercise companionship.

## 1 Introduction

Physical activity is a major public-health concern, but it is also an everyday social practice. Insufficient activity contributes to morbidity, mortality, and economic burden, and global surveillance shows that inactivity remains a population-level problem rather than a marginal behavior (1–6). This public-health evidence establishes why exercise matters, but it does not fully explain how leisure-time exercise is organized in everyday social life.

Research on physical-activity correlates has moved beyond simple individual-choice models. Demographic, socioeconomic, social, and environmental correlates shape activity, and leisure-time, transport-related, and occupational activity follow different social gradients (7–12). Social participation is especially relevant for leisure-time activity (13–15). Yet much of this literature still leaves the social organization of leisure companionship under-specified. Exercise may be an individual behavior, but the routines, invitations, settings, and social ties around leisure can also shape whether exercise becomes a regular practice.

This distinction matters because social relations are not interchangeable. Neighborhood contact, generalized trust, perceived fairness, and friend gatherings represent different forms of social connection and different social distances. Broad social-capital measures are valuable, but grouping all social relations under social capital can obscure the concrete relation most closely connected with leisure-time exercise.

A person who reports high generalized trust, a person who interacts with neighbors, and a person who frequently gathers with friends are not necessarily embedded in the same leisure routines.

The Chinese context makes this question especially important. Rapid urbanization, hukou-based stratification, changing consumption patterns, and rising inequality have reshaped access to leisure, sport, and social participation (16–18). Existing Chinese evidence documents physical-activity patterns and socioeconomic disparities in mass sport participation, including CGSS-based research (19–21). These studies show that sport and physical activity in China are socially patterned, but they less often separate friendship-oriented leisure socializing from neighborhood interaction, trust, or fairness.

This study asks a specific research question: is leisure-time exercise among Chinese adults more strongly associated with friend gatherings than with broader social-relational indicators such as neighborhood interaction, generalized trust, and perceived fairness? The argument is deliberately bounded. Friend gatherings do not directly measure exercising with friends, peer influence, or exercise companionship. They measure the frequency of leisure-time gatherings with friends and are used as a proxy for friendship-oriented leisure socializing. The empirical claim is therefore about adjusted co-patterning, not causation or longitudinal change.

The study makes one main contribution and two scope checks. First, it develops a focused theoretical framework that distinguishes friendship-oriented leisure socializing from broader social capital and from formal sport or venue/gym fitness participation. Second, it tests whether friend gatherings are more strongly associated with general leisure-time exercise than neighborhood interaction, trust, or fairness. Third, it examines whether the result is reducible to venue/gym exercise and whether exercise-friendship profiles have descriptive health relevance. These last analyses are supplementary because the CGSS repeated cross-sectional design cannot identify causal health returns or exercise-friendship synergy.

## 2 Theoretical framework and conceptual boundaries

### 2.1 Conceptual definitions and study scope

Leisure-time exercise is defined empirically by the CGSS PA01 item asking how often respondents participated in physical exercise during leisure time in the past year. It is therefore broader than formal, organized sport and broader than venue/gym exercise. Formal sport typically involves organized rules, teams, clubs, training, or institutional competition. Venue/gym exercise, captured in this study only by PA04 in 2013 and 2023, refers to regular exercise in dedicated sport venues or gyms. General leisure-time exercise may include informal walking, running, dancing, calisthenics, sport, and other non-work physical activity, depending on how respondents understand the survey item.

Friendship-oriented leisure socializing is defined empirically by the CGSS friend-gathering item. The measure indicates how often respondents gather with friends during leisure time. It does not show whether exercise occurred during those gatherings, whether friends encouraged exercise, or whether respondents exercised together. It is used as a proxy for an elective leisure-socializing routine that is more concrete than generalized trust and less place-bound than neighborhood interaction.

This distinction also separates the study from research on community interventions, neighborhood-based initiatives, and team sports. Neighborhood initiatives often depend on place-based access and local infrastructure. Team sports involve organized group participation and sport-specific rules. Friendship-oriented leisure socializing is more selective and relational: it may mark invitations, shared taste, companionship, discretionary time, and active leisure routines. The study examines whether this relational routine co-patterns with exercise more strongly than broader social-capital indicators.

### 2.2 Leisure-time exercise as a socially organized leisure practice

Bourdieu's theory of practice locates everyday behavior at the intersection of resources, dispositions, and social fields (22–24). Health lifestyle theory similarly links life chances and life choices in patterned health practices (25). Research on cultural capital, capabilities, and fundamental causes extends this account (26–28). From this perspective, exercise is not only an individual health behavior. Its organization also depends on time, bodies, facilities, tastes, social circles, and social position.

The same practice perspective places exercise within leisure. Participation depends on constraints, motivations, and available social settings rather than free choice alone (29–31). Parks, streets, land-use mix, and other local opportunities shape walking and recreation (32–36). General leisure-time exercise may therefore form part of wider routines through which people allocate discretionary time and organize social participation.

### 2.3 Friendship-oriented leisure socializing vs. broad social capital

Classic social-capital theories emphasize resources located in networks, norms of reciprocity, civic engagement, trust, and social structure (37–40). Health research has often operationalised social capital through trust, cohesion, participation, or neighborhood ties (41, 42). These concepts are valuable, but they do not represent the same social relation as elective leisure socializing.

Neighborhood interaction can involve proximity, practical help, or community affairs. Generalized trust and perceived fairness are broad social attitudes. Friend gatherings are more concrete, selective, and leisure-oriented.

Friendship patterns also reflect selection and homophily: people tend to form ties with socially similar others, and network behavior can reflect both influence and selection (43–46). In China, personal and strong-tie relations have distinctive significance in job search and everyday exchange (47, 48).

The friend-gathering measure therefore cannot show that friends cause exercise or that respondents exercise together. Repeated cross-sectional data cannot distinguish influence from selection, reverse association, health status, or unobserved dispositions. Co-patterning instead denotes an adjusted association between exercise and friendship-oriented leisure socializing that is stronger than the association between exercise and broader social-capital indicators.

### 2.4 Analytic expectations and scope conditions

The main research question is whether leisure-time exercise among Chinese adults is more strongly associated with friend gatherings than with neighborhood interaction, generalized trust, or perceived fairness.

- H1a. Friend-gathering frequency is positively associated with general leisure-time exercise frequency.
- H1b. The association between friend gatherings and general leisure-time exercise is stronger than the corresponding associations for neighborhood interaction, generalized trust, and perceived fairness.

These expectations concern the relative pattern of adjusted coefficients. They do not specify a causal pathway from friendship to exercise. H1b is evaluated through formal coefficient comparisons rather than visual inspection alone.

Two supplementary analyses define the scope of the main expectation. First, sport-sociology research shows that sport and fitness participation can operate as cultural capital and can be stratified by class (49, 50). A marketised fitness account would anticipate stronger socioeconomic gradients in venue/gym exercise because these practices may require money, access, and cultural familiarity.

The boundary analysis therefore asks whether socioeconomic gradients are consistently stronger for venue/gym exercise than for general exercise. Second, the health-profile analysis assesses public-health relevance, but it does not test causal health returns or friendship-exercise synergy.

## 3 Data and methods

### 3.1 Data source and repeated cross-sectional design

The analysis uses pooled repeated cross-sectional CGSS data from 2010, 2011, 2012, 2013, 2015, 2017, 2018, 2021, and 2023. The CGSS is a major national social survey programme in China. Earlier design documentation describes its multistage national sampling strategy and quality-control goals (19). The pooled working file contains 96,417 person-wave observations before listwise deletion for specific models. The main PA01 variable is observed for 91,833 respondents across the pooled file.

The phrase repeated cross-sectional is used in its survey-design sense: each wave provides a cross-sectional sample, and respondents are anonymous across waves. The data do not follow the same individuals over time. The design therefore supports comparisons of adjusted associations across pooled survey waves, but it does not support individual-level longitudinal inference, within-person change models, or causal claims about friendship leading to exercise.

### 3.2 Measures and descriptive statistics

The main outcome is general leisure-time exercise (PA01), measured by how often respondents participated in physical exercise in their leisure time during the past year. The core predictor is the frequency of friend gatherings. Both are reverse-coded so that higher values indicate more frequent exercise or gatherings. The social-contrast measures are neighborhood interaction, generalized trust, and perceived fairness. The boundary outcome is venue/gym exercise (PA04), available in 2013 and 2023. Socioeconomic indicators include education, personal income, household income, and non-agricultural hukou status. Personal and household income are log-transformed. Demographic controls include female, age, and age squared. Table 1 summarizes the focal constructs, and Table 2 reports descriptive statistics for the main variables.

**Table 1:** Measures and operationalization.

Construct	Item and raw responses	Waves	Harmonization and use
General leisure-time exercise (PA01)	How often respondents participated in physical exercise during leisure time: daily, several times a week, several times a month, several times a year or less, never	2010, 2011, 2012, 2013, 2015, 2017, 2018, 2021, 2023	Reverse-coded 1–5; higher values indicate more frequent exercise. High exercise in Supplementary Table 7 is weekly or more often.
Friend gatherings	How often respondents gathered with friends during leisure time: daily, several times a week, several times a month, several times a year or less, never	2010, 2011, 2012, 2013, 2015, 2017, 2018, 2021, 2023	Reverse-coded 1–5; higher values indicate more frequent gatherings. High friendship in Supplementary Table 7 is weekly or more often.
Neighborhood interaction	Frequency of interaction with neighbors, using the repeated CGSS neighbor-contact item	2011, 2012, 2013, 2015, 2017, 2018, 2021, 2023	Reverse-coded 1–7; higher values indicate more frequent interaction. Used as a contrasting social-relation dimension.
Generalized trust	Agreement that most people in society can be trusted; response categories vary slightly across waves	2010, 2011, 2012, 2013, 2015, 2017, 2018, 2021, 2023	Harmonized so higher values indicate greater trust. Used as a contrasting generalized social-attitude dimension.
Perceived fairness	Perception that society is fair	2010, 2011, 2012, 2013, 2015, 2017, 2018, 2021, 2023	Coded 1–5; higher values indicate greater perceived fairness. Used as a contrasting generalized social-attitude dimension.
Venue/gym exercise (PA04)	Agreement with regularly exercising in dedicated sport venues or gyms: very consistent, somewhat consistent, not very consistent, not consistent	2013 and 2023	Reverse-coded 1–4; higher values indicate stronger venue/gym exercise habit. Used only as a boundary outcome.
Health and wellbeing	Happiness, fewer depressive feelings, and self-rated health	Available waves vary by outcome	Coded 1–5; higher values indicate better outcome or fewer depressive feelings. Used for supplementary profile analysis.

Refused, do-not-know, not-applicable, and out-of-range special codes are treated as missing. The 2023 PA01 analytic sample is smaller because the item is available in the relevant 2023 questionnaire subset.

**Table 2:** Descriptive statistics for main variables.

Variable	*N*	Mean	SD	Min	Max
General leisure-time exercise (PA01)	91,833	2.38	1.54	1	5
Friend gatherings	91,903	2.33	0.99	1	5
Neighborhood interaction	77,692	4.12	2.20	1	7
Generalized trust	90,768	3.45	1.02	1	5
Perceived fairness	91,724	3.14	1.05	1	5
Education	96,289	5.21	3.28	1	14
Personal income, log	88,389	8.43	3.60	0	16
Household income, log	84,215	10.36	1.82	0	16
Non-agricultural hukou	94,085	0.37	0.48	0	1
Female	96,417	0.51	0.50	0	1
Age	96,310	49.56	16.68	18	99
Happiness	93,526	3.85	0.84	1	5
Fewer depressive feelings	90,555	3.84	1.00	1	5
Self-rated health	90,748	3.54	1.09	1	5

Descriptive statistics are calculated from the pooled working file before model-specific listwise deletion. Income variables

### 3.3 Analytic strategy and model-to-question mapping

Province identifiers are harmonized before modeling so that numeric province codes and named province fields refer to the same province fixed effects and clusters; Shenzhen is coded with Guangdong. All regression models include survey-year fixed effects and harmonized province fixed effects. The third main model additionally controls other leisure activities, including television or video watching, movies, shopping, reading, music, and internet use.

The analytic strategy is matched to the research questions. M1 tests the baseline adjusted association between friend gatherings and general leisure-time exercise. M2 adds neighborhood interaction, generalized trust, and perceived fairness and is the primary model for H1b because it allows formal coefficient comparisons among the social-relational predictors. M3 adds other leisure activities as a conservative sensitivity model; these activities may belong to the same active-leisure configuration rather than operate as ordinary confounders. The main models use standardized OLS because the study focuses on association patterns and coefficient comparisons across predictors measured on different scales. The components corresponding to Friend, Neighbor, Trust, and Fairness are compared using the full clustered variance-covariance matrix.

The corresponding generic social-contrast specification is shown in Equation 1.


$$\begin{array}{c} z\left({PA01}_{ipt}\right)=\alpha \;+\;\beta^{T}S_{ipt}\;+\;\gamma^{T}C_{ipt}\;+\;\lambda_{t}\;+\;\mu_{p}\;+\;\varepsilon_{ipt} \end{array}$$ (1)

Here, i indexes respondents, p provinces, and t survey years. z(.) denotes standardization within each model-specific complete-case sample. Sipt is the standardized focal-predictor vector: Friend in M1 and Friend, Neighbor, Trust, and Fairness in M2-M3. Cipt is the standardized control vector; M3 additionally includes the other-leisure measures. Year and province fixed-effect indicators are not standardized. The components corresponding to Friend, Neighbor, Trust, and Fairness are compared using the full clustered variance-covariance matrix.

The boundary analysis uses a fully interacted stacked long-format model for respondents in 2013 and 2023 with both PA01 and PA04 observed. Outcome type equals 1 for PA04 venue/gym exercise and 0 for PA01 general exercise. The interaction terms test whether venue/gym exercise has stronger socioeconomic gradients than general exercise. A conservative difference-score check first standardizes PA04 and PA01 separately, calculates the standardized difference, and regresses that difference on the same socioeconomic and demographic covariates with survey-year and province fixed effects.

The stacked boundary model is shown in Equation 2.


$$\begin{array}{l} z_{j}\left(Y_{ijpt}\right)=\alpha \;+\;\theta V_{j}\;+\;\beta^{T}W_{ipt}\;+\;\varphi^{T}\left(W_{ipt}V_{j}\right)\;+\;\lambda_{t}\;+\;\eta_{t}V_{j}\;+\;\mu_{p}\\ \;+\;\omega_{p}V_{j}\;+\;\varepsilon_{ijpt} \end{array}$$ (2)

Vj equals 1 for PA04 and 0 for PA01, while zj(.) denotes separate standardization within each outcome type. Wipt is the vector of standardized socioeconomic and demographic covariates. The relevant interaction components test whether venue/gym exercise has stronger socioeconomic gradients than general exercise. Demographic controls, survey-year fixed effects, and province fixed effects are allowed to differ by outcome type.

The conservative difference-score check is defined in Equation 3.


$$\begin{array}{c} D_{ipt}=z\left\{z\left({PA04}_{ipt}\right)\;-\;z\left({PA01}_{ipt}\right)\right\} \end{array}$$ (3)

It is then estimated using Equation 4.


$$\begin{array}{c} D_{ipt}=\alpha \;+\;\kappa^{T}W_{ipt}\;+\;\lambda_{t}+\mu_{p}\;+\;\varepsilon_{ipt} \end{array}$$ (4)

Thus, PA04 and PA01 are first standardized separately, their difference is calculated, and the difference score is standardized before estimation. The model includes survey-year and province fixed effects with harmonized-province-clustered standard errors.

The supplementary health-profile model classifies respondents by high vs. low exercise and high vs. low friend gatherings, where high means weekly or more often. This model is reported in the Supplementary material because it describes health and wellbeing profiles but cannot identify causal health returns.

The supplementary health-profile model is shown in Equation 5.


$$\begin{array}{c} z\left(H_{ipt}\right)=\alpha \;+\;\rho^{T}P_{ipt}\;+\;\gamma^{T}{SES}_{ipt}\;+\;\delta^{T}X_{ipt}\;+\;\lambda_{t}\;+\;\mu_{p}\;+\;\varepsilon_{ipt} \end{array}$$ (5)

Hipt denotes happiness, fewer depressive feelings, or self-rated health. Pipt contains the standardized profile indicators, with the low-exercise/low-friendship profile omitted. SESipt and Xipt are the standardized socioeconomic and demographic vectors. Because the profile indicators are standardized before estimation, each profile coefficient is interpreted relative to the omitted profile rather than as an unadjusted mean difference.

### 3.4 Robustness, missing data, and reproducibility checks

The main estimates use harmonized-province-clustered standard errors for single-outcome models and respondent-clustered standard errors for the stacked boundary model. Cluster-robust inference is used because province-level clustering can otherwise understate uncertainty, especially with fixed effects and repeated observations within clusters (51, 52). For province-clustered models, statistical significance is assessed using a t reference distribution with cluster degrees of freedom G-1.

Robustness analyses check whether the main finding depends on the 1-5 exercise scale, the linear functional form, the 2023 questionnaire subset, covariate adjustment, usable survey weights, subgroup composition, single waves, or conventional clustered inference. Supplementary analyses include a binary weekly-exercise model, an ordered-logit model (53), no-2023 models, additional controls for marital status, current work status, and self-rated health, weighted sensitivity models, subgroup checks, leave-one-wave-out models, and wild cluster bootstrap inference. Cross-wave weight construction differs across CGSS waves, so weighted analyses are treated as sensitivity checks rather than the primary estimator (54, 55).

Supplementary Table 1 reports the sample flow from the pooled CGSS file to each model-specific analytic sample. Supplementary Table 2 reports variable-level availability and missingness in the pooled working file. Together with the robustness supplementary tables, these Supplementary materials document the analytic samples, variable availability, missingness, and sensitivity checks used to assess the reported findings. Listwise deletion produces different sample sizes across specifications because variables are unavailable in particular waves or contain item-level missingness.

## 4 Results

### 4.1 Analytic samples and descriptive patterns

The pooled working file contains 96,417 person-wave observations, of which 91,833 have observed PA01. The M1, M2, and M3 analytic samples contain 74,834; 59,960, and 59,596 observations, respectively. Descriptively, the pooled mean of general leisure-time exercise is 2.38 (SD = 1.54) on the harmonized 1-5 scale, while the pooled mean of friend gatherings is 2.33 (SD = 0.99). Neighborhood interaction has a mean of 4.12 (SD = 2.20) on the harmonized 1-7 scale, and the pooled mean age is 49.56 years (SD = 16.68). The exercise mean rises from 2.11 in 2010 to 2.72 in 2023, although the 2023 value requires caution because PA01 is observed only in the relevant questionnaire subset. Friend gatherings are comparatively stable, from 2.27 in 2010 to 2.32 in 2023.

### 4.2 Main social-relational contrast

**Figure 1:**
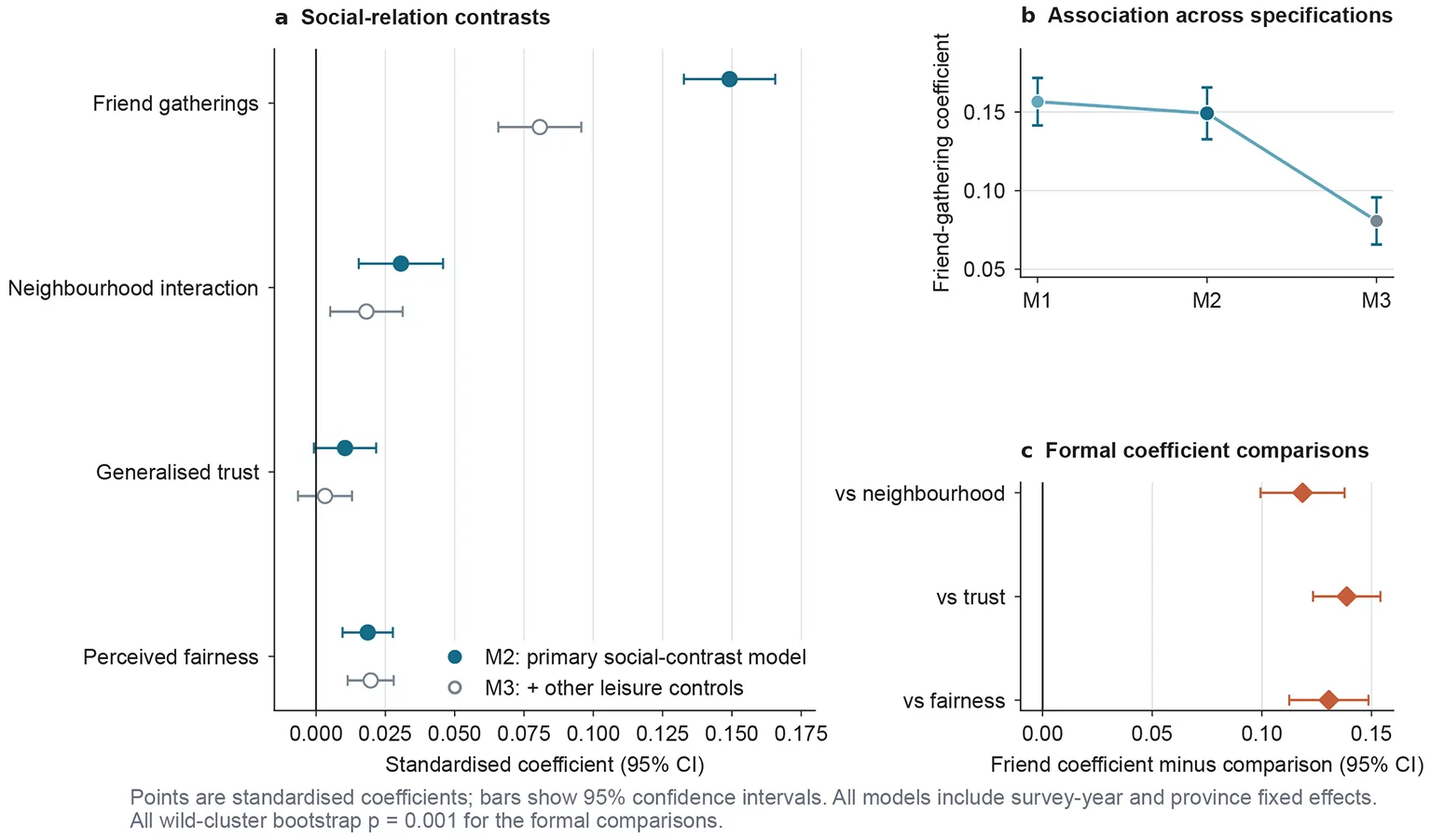
Friendship-oriented co-patterning and the main social contrast. Points are standardized coefficients and bars are 95% confidence intervals. M2 is the primary social-contrast model; M3 additionally adjusts for other leisure activities. The formal differences compare the friend-gathering coefficient with each alternative social-relation coefficient. **(a)** Social-relation contrasts; **(b)** Association across specifications; **(c)** Formal coefficient comparisons.

Table 3 and Figure 1 present the main test of the friendship-oriented co-patterning expectation. In M1, friend gatherings are positively associated with general exercise after adjustment for SES, demographics, survey-year fixed effects, and harmonized province fixed effects (standardized beta = 0.157, *p* < 0.001). The coefficient remains similar in M2 after adding neighborhood interaction, generalized trust, and perceived fairness (beta = 0.149, *p* < 0.001). The neighborhood coefficient is 0.031, the generalized trust coefficient is 0.010, and the perceived fairness coefficient is 0.019. Formal tests show that the friend-gathering coefficient exceeds the neighborhood coefficient (difference = 0.119, *p* < 0.001). It also exceeds the trust coefficient (difference = 0.139, *p* < 0.001) and fairness coefficient (difference = 0.131, *p* < 0.001). Supplementary Table 3 reports the numerical tests. Generalized trust is not statistically significant under harmonized province clustering and small-cluster inference.

**Table 3:** Friendship-oriented co-patterning of general leisure-time exercise.

Predictor	M1	M2	M3
Friend gatherings	0.157 (0.008)^***^	0.149 (0.008)^***^	0.081 (0.008)^***^
Neighborhood interaction		0.031 (0.008)^***^	0.018 (0.007)^*^
Generalized trust		0.010 (0.006)	0.003 (0.005)
Perceived fairness		0.019 (0.005)^***^	0.020 (0.004)^***^
Education	0.199 (0.017)^***^	0.200 (0.019)^***^	0.087 (0.013)^***^
Personal income, log	−0.015 (0.006)^*^	−0.014 (0.007)	−0.020 (0.006)^**^
Household income, log	0.063 (0.006)^***^	0.059 (0.005)^***^	0.029 (0.005)^***^
Non-agricultural hukou	0.130 (0.011)^***^	0.133 (0.011)^***^	0.081 (0.010)^***^
Female	−0.013 (0.006)^*^	−0.015 (0.006)^*^	−0.030 (0.008)^***^
Age	0.157 (0.048)^**^	0.127 (0.050)^*^	0.315 (0.041)^***^
Age squared	−0.051 (0.041)	−0.036 (0.045)	−0.161 (0.038)^***^
*N*	74,834	59,960	59,596

Entries are standardized OLS coefficients with harmonized-province-clustered standard errors in parentheses. Province identifiers are harmonized across numeric and named CGSS fields before fixed effects and clustering. Significance uses a t reference distribution with cluster degrees of freedom G-1. All models include survey-year and province fixed effects. M1 controls SES and demographics; M2 is the primary social-contrast model; M3 additionally controls television, movies, shopping, reading, music, and internet use. ^*^ *p* < 0.05, ^**^ *p* < 0.01, ^***^ *p* < 0.001.

### 4.3 Lifestyle-adjusted and robustness analyses

M3 is a conservative lifestyle-adjusted sensitivity model. The friend-gathering coefficient falls from 0.149 in M2 to 0.081 in M3, a 45.9% reduction based on the unrounded coefficients. This attenuation should not be interpreted as simple confounding control. The additional variables may belong to the same leisure-practice configuration rather than sit outside the phenomenon. Friend gatherings therefore mark a wider leisure pattern while retaining an adjusted association not captured by the included leisure measures.

Excluding the 2023 questionnaire subset produces similar coefficients: 0.156 in M1, 0.150 in M2, and 0.081 in M3. Period-specific M2 estimates remain positive before 2020 (beta = 0.150, *p* < 0.001) and after 2020 (beta = 0.149, *p* < 0.001). The result is also positive in binary weekly-exercise models (M2 beta = 0.119; M3 beta = 0.063) and in ordered-logit models (M2 coefficient = 0.355; M3 coefficient = 0.208). All four sensitivity estimates have *p* < 0.001.

**Figure 2:**
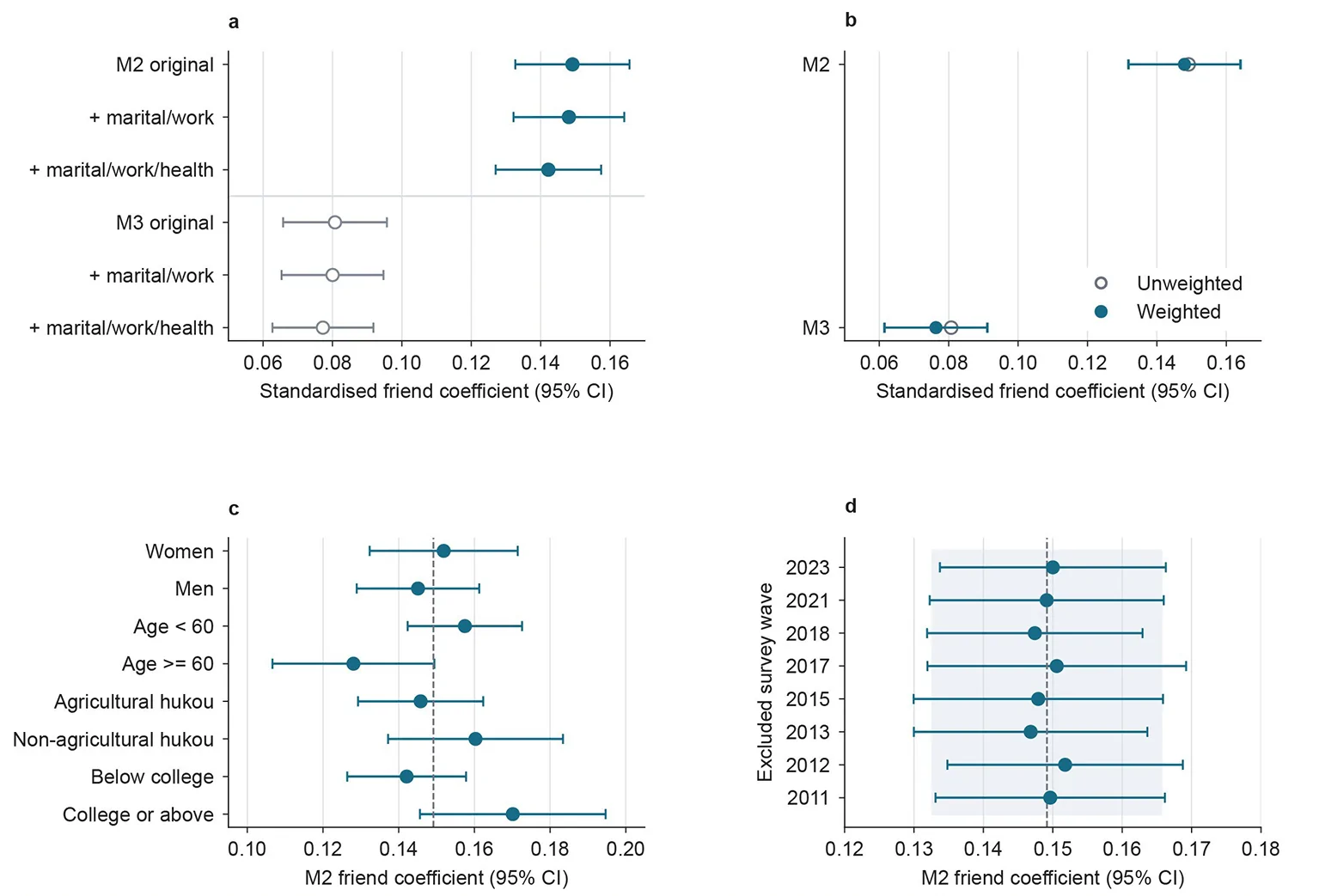
Robustness of the friend-gathering association. Note. All panels report the friend-gathering coefficient. Bars are 95% confidence intervals. Dashed lines and the shaded band mark the pooled M2 estimate and its 95% confidence interval. **(a)** Additional controls; **(b)** Survey-weight sensitivity; **(c)** Subgroup consistency; **(d)** Leave-one-wave-out estimates.

Additional robustness analyses are summarized in Figure 2 and Supplementary Tables 8–12. Adding marital and employment controls leaves the M2 coefficient essentially unchanged (beta = 0.148, *p* < 0.001). Adding self-rated health reduces it modestly to 0.142 (*p* < 0.001). Weighted estimates are close to unweighted estimates (M2 beta = 0.148; M3 beta = 0.076). Friend coefficients are positive in every prespecified subgroup, leave-one-wave-out estimates range from 0.147 to 0.152, and wild cluster bootstrap *p* values are 0.0010 for the friend coefficient and each M2 comparison. These results are consistent with both components of the main analytic expectation.

### 4.4 Boundary and supplementary health-profile analyses

**Figure 3:**
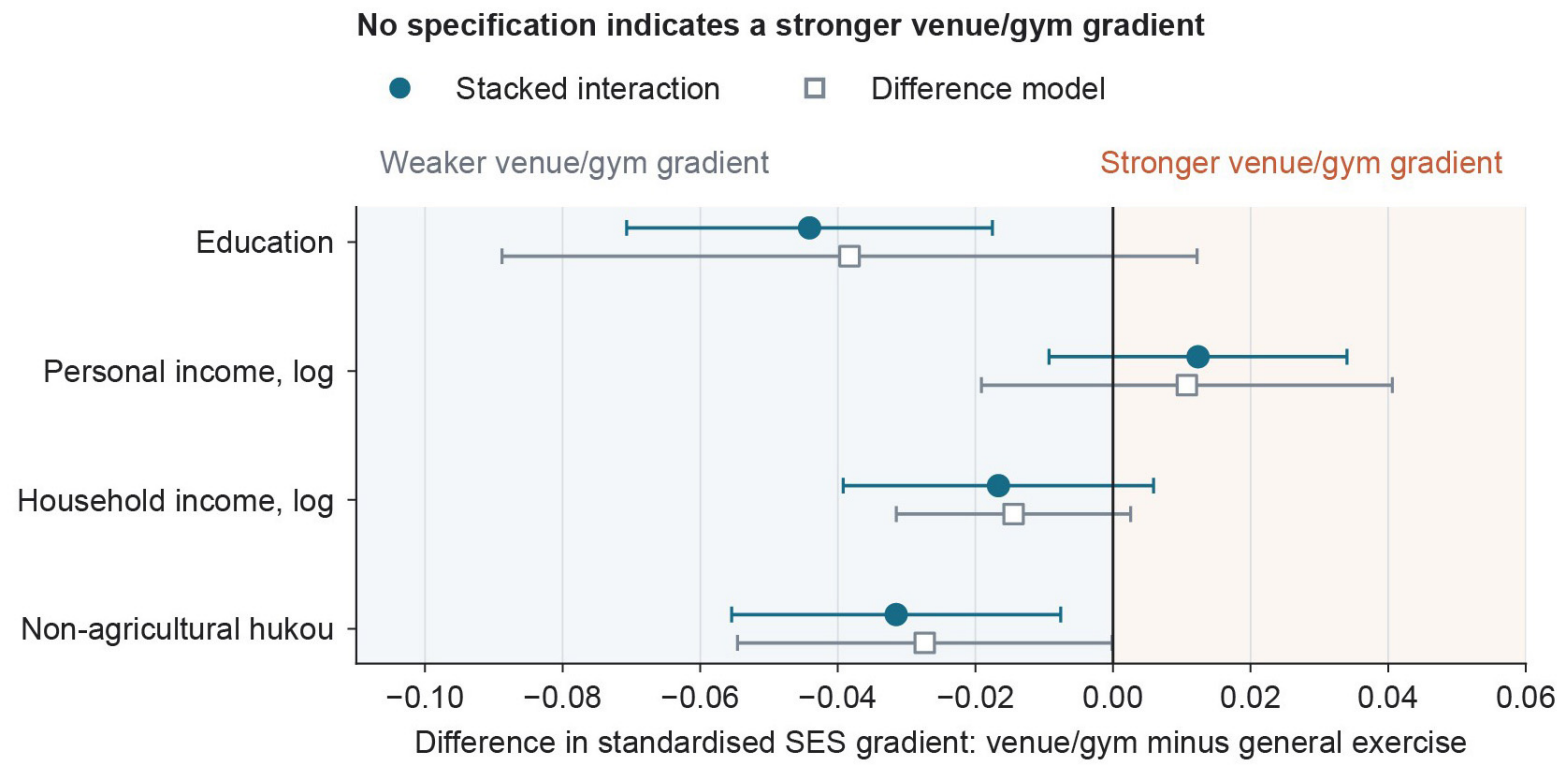
Boundary test for venue/gym exercise. Points show differential standardized socioeconomic gradients for PA04 venue/gym exercise relative to PA01 general exercise; bars are 95% confidence intervals. Positive estimates would indicate a stronger venue/gym gradient.

Table 4 and Figure 3 present a supplementary boundary test using two specifications. PA04 is available only in 2013 and 2023 and measures agreement with venue/gym exercise. The analysis is therefore interpreted as a scope-condition check. The stacked model provides no evidence that venue/gym exercise is more strongly stratified by income than general exercise. The PA04 interactions are not significant for personal income (beta = 0.012, *p* = 0.264) or household income (beta = −0.017, *p* = 0.148).

**Table 4:** Boundary test: general exercise vs. venue/gym exercise.

SES indicator	Stacked interaction beta	Difference-model beta	Conservative interpretation
Education	−0.044 (0.014)^**^	−0.038 (0.026)	Weaker in stacked model; not stronger in conservative check
Personal income, log	0.012 (0.011)	0.011 (0.015)	No evidence that the venue/gym gradient is stronger
Household income, log	−0.017 (0.012)	−0.014 (0.009)	No evidence that the venue/gym gradient is stronger
Non-agricultural hukou	−0.032 (0.012)^**^	−0.027 (0.014)	Weaker in stacked model; not stronger in conservative check

Stacked entries are standardized interaction coefficients from a fully interacted stacked long-format model restricted to respondents in 2013 and 2023 with both PA01 and PA04 observed. Outcome type is coded 1 for PA04 venue/gym exercise and 0 for PA01 general exercise. Negative values indicate a weaker venue/gym gradient than general-exercise gradient. The difference-model column regresses standardized PA04 minus standardized PA01 on the same SES and demographic controls with survey-year and province fixed effects, using harmonized-province-clustered standard errors and G - 1 cluster degrees of freedom.

Education and hukou interactions are negative in the stacked model. Their coefficients are −0.044 (*p* = 0.001) and −0.032 (*p* = 0.010), respectively. The province-clustered difference model weakens these findings. Education is −0.038 (*p* = 0.148), and hukou is −0.027 (*p* = 0.058). The conservative conclusion is that the main result is not obviously reducible to a venue/gym-exercise gradient.

The supplementary health-profile analysis is reported in Supplementary Table 7 and Supplementary Figure 1. Descriptively, the high-exercise/high-friendship group has the most favorable means for happiness, fewer depressive feelings, and self-rated health. In adjusted models, however, the high-exercise/high-friendship coefficients remain positive relative to the low-exercise/low-friendship reference group but are smaller than the high-exercise/low-friendship coefficients. Interaction checks also do not support a synergy interpretation. The profile analysis therefore suggests public-health relevance of exercise and friendship as components of active leisure, but it does not support additional health returns from their combination.

## 5 Discussion

### 5.1 Principal interpretation

The findings support the central claim that leisure-time exercise among Chinese adults is more strongly associated with friendship-oriented leisure socializing than with neighborhood interaction, generalized trust, or perceived fairness. This does not mean that neighborhood ties, trust, or fairness are irrelevant. It means that the social basis of exercise in these data is more concrete, elective, and leisure-oriented than a broad social-capital explanation would suggest.

Theoretically, the results refine social-capital interpretations of physical activity. Exercise is more closely aligned with friendship-oriented leisure socializing than with generalized trust or neighborhood connectedness in these data. This pattern is consistent with Bourdieusian and health-lifestyle accounts of exercise as a practice shaped by social position and routine. Exercise may form part of a socially patterned way of living, not only an individual health behavior. The construct-validity boundary remains central. The evidence identifies adjusted co-patterning between exercise and friend gatherings, not a mechanism of exercising with friends.

### 5.2 Boundary conditions and public-health relevance

The attenuation after adding other leisure activities is theoretically meaningful. Friend gatherings are not an isolated predictor. They are part of a broader leisure-practice configuration that also includes reading, music, shopping, internet use, and other discretionary practices. Because these variables may belong to the same lifestyle structure, M3 should be read as a conservative sensitivity model rather than the sole preferred specification. Its positive coefficient indicates that the included leisure measures do not fully account for the association between friend gatherings and exercise.

The added robustness checks strengthen descriptive confidence without changing the claim's causal status. The association remains positive after additional controls and usable survey weighting. It is also positive across sex, age, hukou, and education subgroups, and after omitting each wave. Wild cluster bootstrap inference supports the main coefficient and comparison tests. These checks reduce sensitivity to one covariate set, wave, or conventional clustered inference. They do not resolve selection or unobserved heterogeneity.

The boundary test adds a more limited implication. Neither specification shows stronger socioeconomic gradients for venue/gym exercise than for general exercise. The friendship-oriented result is therefore not obviously reducible to a venue/gym-exercise gradient. This does not show that marketised fitness consumption is irrelevant. Nor does it constitute a complete analysis of physical-activity stratification. Sport-sociology research similarly shows that class and cultural capital shape some forms of participation (49, 50). Other forms of active leisure may be socially organized outside commercial venues.

The health-relevance results should be read with equal caution. Physical activity is consistently associated with better physical and mental health in prior research (56–59). In this study, the high-exercise/high-friendship group has the most favorable descriptive means, but adjusted models suggest that health-related differences are driven more strongly by exercise than by friendship. The evidence therefore does not show additional health returns from combining frequent exercise with frequent friend gatherings.

### 5.3 Policy implications

The policy implications are hypothesis-generating rather than intervention evidence. The findings do not show that a specific programme would increase exercise. They suggest instead that future physical-activity research should test social organization alongside physical infrastructure. Prior intervention research supports multilevel approaches that combine environments, programmes, and social participation (60–63). Walking groups, community sport teams, dance groups, and referral-based programmes should therefore be treated as candidate approaches for future evaluation rather than as interventions supported directly by the present estimates.

Such programmes should be tested for accessibility and heterogeneous uptake rather than assumed to work uniformly. Relevant design features include low fees, predictable schedules, accessible spaces, and options for people who do not arrive with an established activity network. The present estimates cannot determine which design is effective or which population would benefit most.

### 5.4 Limitations and future research

Several limitations define the interpretation. First, the CGSS data are repeated cross-sections. Respondents are anonymous across waves, so the data cannot establish whether friend gatherings cause exercise, exercise affects health, or active-leisure profiles produce wellbeing. Selection and influence are difficult to separate even longitudinally and cannot be separated here (45). Second, the measures are self-reported and harmonized across waves, which introduces measurement error. Friend gatherings proxy friendship-oriented leisure socializing rather than exercise companionship.

Third, standardized OLS estimates describe association patterns, not population prevalence. Fourth, cross-wave comparability of usable survey weights remains imperfect. Fifth, PA01 is observed only in the relevant 2023 questionnaire subset. Excluding 2023 yields similar main estimates, but survey-mode and subset differences remain. Sixth, the PA04 boundary test is limited to 2013 and 2023. Claims that some venue/gym gradients are weaker are specification-sensitive. Finally, robustness analyses cannot remove selection, homophily, unobserved personality, or unobserved health differences.

These limitations also define the future research agenda. A primary survey designed specifically for this topic would be valuable because it could measure friendship quality, exercise companionship, solitary vs. co-participatory exercise, formal sport participation, fitness settings, and both physical and mental health outcomes. Such data would allow stronger tests of mechanisms than are possible with CGSS secondary data. The present study should therefore be read as a nationally repeated, secondary-data association study that identifies a theoretically meaningful co-patterning, not as a substitute for future primary survey or longitudinal work.

### 5.5 Conclusion

Leisure-time exercise among Chinese adults co-patterns with friendship-oriented leisure socializing. Across CGSS waves from 2010 to 2023, friend gatherings are more strongly associated with exercise than are neighborhood interaction, generalized trust, or perceived fairness. Formal coefficient comparisons and sensitivity analyses support this empirical contrast. Supplementary analyses do not show that the pattern is reducible to venue/gym exercise or that exercise and friend gatherings produce synergistic health benefits. The contribution is therefore bounded but substantive: physical activity is associated with concrete everyday leisure socializing as well as individual and socioeconomic characteristics.

## Data Availability

Publicly available datasets were analyzed in this study. The data were obtained from the Chinese General Social Survey (CGSS), conducted by the National Survey Research Center at Renmin University of China. The datasets are available through the China National Social Survey Data Archive (CNSSDA): https://www.cnsda.org/index.php. No accession number is applicable. Access may require user registration and compliance with the data-use requirements of the data provider.
