# Supplementary material for: Association between friend gatherings and leisure-time exercise among Chinese adults
Source: Front Public Health. 2026 Aug 7;14:1909863. doi: 10.3389/fpubh.2026.1909863 (PMC13494600; doi:10.3389/fpubh.2026.1909863)
Supplement: Supplementary file 1 [file Table_1.docx]

Supplementary Material

# 1 Supplementary tables and figure

This file contains the supplementary analyses referenced in the main manuscript. All tables are editable, and the supplementary figure is included with its caption.

## 1.1 Supplementary Table 1. Analytic Sample Flow

| **Step** | **N** | **Loss** | **Province clusters** | **Reason** |
| --- | --- | --- | --- | --- |
| Pooled CGSS 2010-2023 working file | 96,417 | -- | 31 | Initial pooled repeated cross-sectional working file. |
| PA01 observed | 91,833 | 4,584 | 31 | General leisure-time exercise observed; special missing and inapplicable codes removed. |
| M1 analytic sample | 74,834 | 16,999 | 31 | Complete cases for PA01, friend gatherings, SES, demographics, survey year, and harmonised province. |
| M2 analytic sample | 59,960 | 14,874 | 29 | Adds neighbourhood interaction, generalised trust, and perceived fairness; neighbourhood interaction is unavailable in 2010 and item-level missingness remains. |
| M3 analytic sample | 59,596 | 364 | 29 | Adds complete information on television/video watching, movies, shopping, reading, music, and internet use. |

Note. Losses are relative to the previous row. Province clusters are harmonised before fixed effects and clustering; cluster counts vary across models because listwise deletion is applied separately by specification and because some variables are unavailable or sparsely observed in specific survey waves.

## 1.2 Supplementary Table 2. Variable Availability and Missingness

| **Variable** | **Available waves** | **Nonmissing N** | **Missing %** | **Enters** |
| --- | --- | --- | --- | --- |
| PA01 general exercise | 2010, 2011, 2012, 2013, 2015, 2017, 2018, 2021, 2023 | 91,833 | 4.8 | M1-M3 |
| Friend gatherings | 2010, 2011, 2012, 2013, 2015, 2017, 2018, 2021, 2023 | 91,903 | 4.7 | M1-M3 |
| Neighbourhood interaction | 2011, 2012, 2013, 2015, 2017, 2018, 2021, 2023 | 77,692 | 19.4 | M2-M3 |
| Generalised trust | 2010, 2011, 2012, 2013, 2015, 2017, 2018, 2021, 2023 | 90,768 | 5.9 | M2-M3 |
| Perceived fairness | 2010, 2011, 2012, 2013, 2015, 2017, 2018, 2021, 2023 | 91,724 | 4.9 | M2-M3 |
| Education | 2010, 2011, 2012, 2013, 2015, 2017, 2018, 2021, 2023 | 96,289 | 0.1 | M1-M3 |
| Personal income, log | 2010, 2011, 2012, 2013, 2015, 2017, 2018, 2021, 2023 | 88,389 | 8.3 | M1-M3 |
| Household income, log | 2010, 2011, 2012, 2013, 2015, 2017, 2018, 2021, 2023 | 84,215 | 12.7 | M1-M3 |
| Non-agricultural hukou | 2010, 2011, 2012, 2013, 2015, 2017, 2018, 2021, 2023 | 94,085 | 2.4 | M1-M3 |
| Female | 2010, 2011, 2012, 2013, 2015, 2017, 2018, 2021, 2023 | 96,417 | 0.0 | M1-M3 |
| Age | 2010, 2011, 2012, 2013, 2015, 2017, 2018, 2021, 2023 | 96,310 | 0.1 | M1-M3 |
| Television/video watching | 2010, 2011, 2012, 2013, 2015, 2017, 2018, 2021, 2023 | 91,889 | 4.7 | M3 |
| Movies | 2010, 2011, 2012, 2013, 2015, 2017, 2018, 2021, 2023 | 91,857 | 4.7 | M3 |
| Shopping | 2010, 2011, 2012, 2013, 2015, 2017, 2018, 2021, 2023 | 91,857 | 4.7 | M3 |
| Reading | 2010, 2011, 2012, 2013, 2015, 2017, 2018, 2021, 2023 | 91,829 | 4.8 | M3 |
| Music | 2010, 2011, 2012, 2013, 2015, 2017, 2018, 2021, 2023 | 91,867 | 4.7 | M3 |
| Internet use | 2010, 2011, 2012, 2013, 2015, 2017, 2018, 2021, 2023 | 91,869 | 4.7 | M3 |
| PA04 venue/gym exercise | 2013, 2023 | 18,397 | 80.9 | Boundary test |

Note. Percentages use the pooled working file as the denominator. PA04 is listed for transparency but is used only in the 2013/2023 boundary analysis, not in M1-M3.

## 1.3 Supplementary Table 3. Formal Coefficient Comparisons in M2

| **Comparison** | **Difference** | **SE** | **p** | **N** | **Clusters** |
| --- | --- | --- | --- | --- | --- |
| Friend - neighbourhood interaction | 0.119 | 0.010 | <.001 | 59,960 | 29 |
| Friend - generalised trust | 0.139 | 0.008 | <.001 | 59,960 | 29 |
| Friend - perceived fairness | 0.131 | 0.009 | <.001 | 59,960 | 29 |

Note. Tests use the M2 social-contrast model and evaluate whether the standardised friend-gathering coefficient differs from each contrasting social-relation coefficient. Standard errors are computed from the harmonised-province-clustered variance-covariance matrix, with p values evaluated using cluster degrees of freedom G - 1.

## 1.4 Supplementary Table 4. Period-Specific Friend-Gathering Estimates

| **Sample** | **Friend β** | **SE** | **p** | **N** | **Clusters** |
| --- | --- | --- | --- | --- | --- |
| Pre-2020 waves | 0.150 | 0.009 | <.001 | 52,989 | 29 |
| Post-2020 waves | 0.149 | 0.018 | <.001 | 6,971 | 25 |

Note. Period-specific estimates use the M2 social-contrast specification.

## 1.5 Supplementary Table 5. Binary Weekly-Exercise Robustness

| **Model** | **Friend β** | **SE** | **p** | **N** | **Clusters** |
| --- | --- | --- | --- | --- | --- |
| Binary weekly-exercise M2 | 0.119 | 0.008 | <.001 | 59,960 | 29 |
| Binary weekly-exercise M3 | 0.063 | 0.007 | <.001 | 59,596 | 29 |

Note. The outcome is a binary indicator for weekly-or-more general leisure-time exercise (PA01 values 4-5 after recoding). Entries are standardised linear probability model estimates with harmonised-province-clustered standard errors and cluster degrees of freedom G - 1.

## 1.6 Supplementary Table 6. Ordered-Logit Robustness

| **Model** | **Friend logit coef.** | **SE** | **p** | **N** | **Clusters** |
| --- | --- | --- | --- | --- | --- |
| Ordered logit M2 | 0.355 | 0.018 | <.001 | 59,960 | 29 |
| Ordered logit M3 | 0.208 | 0.017 | <.001 | 59,596 | 29 |

Note. The outcome is the original ordered PA01 general leisure-time exercise scale after harmonisation (1-5, higher values indicate more frequent exercise). Entries are ordered-logit coefficients for standardised predictors, with survey-year fixed effects, harmonised province fixed effects, and harmonised-province-clustered standard errors. P values use cluster degrees of freedom G - 1.

## 1.7 Supplementary Table 7. Supplementary Descriptive Health Relevance of Active-Leisure Profiles

| **Active-leisure profile** | **Happiness mean** | **Happiness adjusted β** | **Fewer depressive feelings mean** | **Fewer depressive feelings adjusted β** | **Self-rated health mean** | **Self-rated health adjusted β** |
| --- | --- | --- | --- | --- | --- | --- |
| Low exercise, low friends | 3.761 | Reference | 3.779 | Reference | 3.462 | Reference |
| High exercise, low friends | 4.014 | 0.091 (0.005)*** | 4.035 | 0.066 (0.005)*** | 3.674 | 0.070 (0.005)*** |
| Low exercise, high friends | 3.904 | 0.036 (0.004)*** | 3.887 | 0.016 (0.004)*** | 3.705 | 0.024 (0.004)*** |
| High exercise, high friends | 4.059 | 0.058 (0.004)*** | 4.060 | 0.033 (0.004)*** | 3.882 | 0.046 (0.003)*** |

Note. High exercise and high friendship mean weekly or more often. Adjusted entries are standardised OLS coefficients with harmonised-province-clustered standard errors in parentheses; significance uses a t reference distribution with cluster degrees of freedom G - 1. The reference group is low exercise and low friend gatherings. The high-exercise/high-friendship group has the most favourable descriptive means, but adjusted differences are stronger for high exercise with low friendship than for high exercise with high friendship.

## 1.8 Supplementary Figure 1. Adjusted Health Differences across Active-Leisure Profiles

Note. The reference group is low exercise and low friend gatherings. High means weekly or more often. Points are adjusted standardised differences and bars are 95% confidence intervals. These estimates are descriptive and do not identify exercise-friendship health synergy.

## 1.9 Supplementary Table 8. Additional-Control Robustness for the Friend-Gathering Coefficient

| **Model** | **Friend β (SE)** | **p** | **N** | **Clusters** |
| --- | --- | --- | --- | --- |
| M2 original | 0.149 (0.008)*** | <.001 | 59,960 | 29 |
| M2 + marital/work controls | 0.148 (0.008)*** | <.001 | 59,936 | 29 |
| M2 + marital/work/self-rated-health controls | 0.142 (0.008)*** | <.001 | 55,441 | 29 |
| M3 original | 0.081 (0.008)*** | <.001 | 59,596 | 29 |
| M3 + marital/work controls | 0.080 (0.007)*** | <.001 | 59,573 | 29 |
| M3 + marital/work/self-rated-health controls | 0.077 (0.007)*** | <.001 | 55,139 | 29 |

Note. Entries are standardised OLS coefficients for friend gatherings. Added controls are marital/partnered status, current work status, and self-rated health. All models include the same fixed effects and clustered inference as the corresponding main model.

## 1.10 Supplementary Table 9. Survey-Weight Sensitivity

| **Model** | **Unweighted β** | **Weighted β** | **Weighted SE** | **Weighted p** | **N** | **Clusters** |
| --- | --- | --- | --- | --- | --- | --- |
| Weighted M2 | 0.149 | 0.148 | 0.008 | <.001 | 59,937 | 29 |
| Weighted M3 | 0.081 | 0.076 | 0.008 | <.001 | 59,573 | 29 |

Note. Weighted models use usable survey weights in the local CGSS extract, with 2023 using the integrated weight. Because cross-wave weight construction differs across CGSS waves, weighted analyses are presented as sensitivity checks rather than the primary estimator. M2 and M3 complete cases exclude 2010 because neighbourhood interaction is unavailable for that wave. The weighted N is slightly lower than the corresponding unweighted N because observations with missing or non-positive usable weights are omitted after applying the same model-specific complete-case requirements.

## 1.11 Supplementary Table 10. Heterogeneity Checks for the M2 Friend-Gathering Coefficient

| **Subgroup** | **Friend β (SE)** | **p** | **N** | **Clusters** |
| --- | --- | --- | --- | --- |
| Women | 0.152 (0.010)*** | <.001 | 30,593 | 29 |
| Men | 0.145 (0.008)*** | <.001 | 29,367 | 29 |
| Age < 60 | 0.157 (0.008)*** | <.001 | 40,752 | 29 |
| Age >= 60 | 0.128 (0.011)*** | <.001 | 19,208 | 29 |
| Agricultural hukou | 0.146 (0.008)*** | <.001 | 38,032 | 29 |
| Non-agricultural hukou | 0.160 (0.012)*** | <.001 | 21,928 | 29 |
| Below college | 0.142 (0.008)*** | <.001 | 50,059 | 29 |
| College or above | 0.170 (0.013)*** | <.001 | 9,901 | 29 |

Note. Entries use the M2 social-contrast specification. The subgroup-defining variable is omitted when it is constant within the subgroup.

## 1.12 Supplementary Table 11. Leave-One-Wave-Out M2 Robustness

| **Excluded wave** | **Friend β (SE)** | **p** | **N** | **Clusters** |
| --- | --- | --- | --- | --- |
| 2011 | 0.150 (0.008)*** | <.001 | 55,476 | 29 |
| 2012 | 0.152 (0.009)*** | <.001 | 50,653 | 28 |
| 2013 | 0.147 (0.009)*** | <.001 | 50,549 | 29 |
| 2015 | 0.148 (0.009)*** | <.001 | 52,212 | 29 |
| 2017 | 0.151 (0.010)*** | <.001 | 48,978 | 29 |
| 2018 | 0.147 (0.008)*** | <.001 | 48,903 | 29 |
| 2021 | 0.149 (0.009)*** | <.001 | 57,937 | 29 |
| 2023 | 0.150 (0.008)*** | <.001 | 55,012 | 29 |

Note. Each row re-estimates the M2 social-contrast model after excluding one survey wave.

## 1.13 Supplementary Table 12. Wild Cluster Bootstrap Inference for M2

| **Test** | **Estimate** | **Cluster t** | **Cluster p** | **Wild p** | **Valid reps** | **N** | **Clusters** |
| --- | --- | --- | --- | --- | --- | --- | --- |
| Friend coefficient | 0.149 | 17.757 | <.001 | 0.0010 | 999 | 59,960 | 29 |
| Friend - neighbourhood interaction | 0.119 | 12.122 | <.001 | 0.0010 | 999 | 59,960 | 29 |
| Friend - generalised trust | 0.139 | 17.714 | <.001 | 0.0010 | 999 | 59,960 | 29 |
| Friend - perceived fairness | 0.131 | 14.155 | <.001 | 0.0010 | 999 | 59,960 | 29 |

Note. Wild cluster bootstrap p values use Rademacher weights at the harmonised-province cluster level with 999 requested replications. Tests evaluate the friend-gathering coefficient and the three M2 coefficient comparisons.
